# Cord blood-derived mesenchymal stromal cells in children with steroid-dependent nephrotic syndrome: a prospective phase II study

**DOI:** 10.1093/ckj/sfag179

**Published:** 2026-05-30

**Authors:** William Morello, Elisa Montelatici, Cristiana Lavazza, Silvia Budelli, Lorenza Lazzari, Giuseppe Puccio, Chiara Bulgaro, Emilio Carrino, Salvatore La Rosa, Chiara Tamburello, Teresa Nittoli, Laura Catenacci, Tiziana Montemurro, Luciana Ghio, Alberto Edefonti, Rosaria Giordano, Giovanni Montini

**Affiliations:** Pediatric Nephrology, Dialysis and Transplant Unit, Fondazione IRCCS Ca' Granda, Ospedale Maggiore Policlinico, Milano, Italy; Unit of Cellular and Gene Therapy, Fondazione IRCCS Ca' Granda Ospedale Maggiore Policlinico, Milano, Italy; Unit of Cellular and Gene Therapy, Fondazione IRCCS Ca' Granda Ospedale Maggiore Policlinico, Milano, Italy; Unit of Cellular and Gene Therapy, Fondazione IRCCS Ca' Granda Ospedale Maggiore Policlinico, Milano, Italy; Unit of Cellular and Gene Therapy, Fondazione IRCCS Ca' Granda Ospedale Maggiore Policlinico, Milano, Italy; Pediatric Nephrology, Dialysis and Transplant Unit, Fondazione IRCCS Ca' Granda, Ospedale Maggiore Policlinico, Milano, Italy; Pediatric Nephrology, Dialysis and Transplant Unit, Fondazione IRCCS Ca' Granda, Ospedale Maggiore Policlinico, Milano, Italy; Unit of Cellular and Gene Therapy, Fondazione IRCCS Ca' Granda Ospedale Maggiore Policlinico, Milano, Italy; Unit of Cellular and Gene Therapy, Fondazione IRCCS Ca' Granda Ospedale Maggiore Policlinico, Milano, Italy; Pediatric Nephrology, Dialysis and Transplant Unit, Fondazione IRCCS Ca' Granda, Ospedale Maggiore Policlinico, Milano, Italy; Pediatric Nephrology, Dialysis and Transplant Unit, Fondazione IRCCS Ca' Granda, Ospedale Maggiore Policlinico, Milano, Italy; Pediatric Hematology Oncology and Cell Factory, Department of Maternal and Children’s Health, Foundation IRCCS Policlinico San Matteo, Pavia, Italy; Unit of Cellular and Gene Therapy, Fondazione IRCCS Ca' Granda Ospedale Maggiore Policlinico, Milano, Italy; Pediatric Nephrology, Dialysis and Transplant Unit, Fondazione IRCCS Ca' Granda, Ospedale Maggiore Policlinico, Milano, Italy; Pediatric Nephrology, Dialysis and Transplant Unit, Fondazione IRCCS Ca' Granda, Ospedale Maggiore Policlinico, Milano, Italy; Unit of Cellular and Gene Therapy, Fondazione IRCCS Ca' Granda Ospedale Maggiore Policlinico, Milano, Italy; Pediatric Nephrology, Dialysis and Transplant Unit, Fondazione IRCCS Ca' Granda, Ospedale Maggiore Policlinico, Milano, Italy; Department of Clinical Sciences and Community Health, Dipartimento di Eccellenza 2023-2027, University of Milan, Italy

**Keywords:** advanced therapy medicinal products, children, cord blood-derived mesenchymal stromal cells, immune-mediated glomerular diseases, immunosuppression, steroid-dependent idiopathic nephrotic syndrome

## Abstract

**Background:**

Children with steroid-dependent nephrotic syndrome (SDNS) require long-term immunosuppressive (IS) treatment, significantly impacting their quality of life. Cord-blood-derived mesenchymal stromal cells (CB-MSCs) are multipotent stem cells with proven immunomodulatory properties.

**Methods:**

We conducted an adaptive, open-label, single-arm, phase II trial with an adaptive second part to evaluate the efficacy of CB-MSCs in children aged 3–18 years with SDNS in remission for ≥6 months on IS therapy. In Part 1, patients received three intravenous infusions of CB-MSCs (1.5 × 10⁶/kg) at 1–2-week intervals, with rapid IS tapering and discontinuation after the third infusion. The primary endpoint was relapse-free survival at 6 months post-IS withdrawal, with a threshold of <4/11 relapsing patients. In the adaptive Part 2, IS was discontinued before the first infusion to enhance CB-MSCs activity, and the dose was increased to 2 × 10⁶/kg, with a booster fourth infusion. A historical cohort of SDNS children tapering IS under standard care served as a post-hoc control.

**Results:**

In Part 1, 9 patients were enrolled, and 7/9 (78%) relapsed, failing to meet the prespecified efficacy threshold. In Part 2, 11 patients were enrolled; 9 (82%) were in remission at 2 weeks post-third infusion and received the boosted dose. However, 6/11 (55%) relapsed within 6 months, with no significant difference in relapse-free survival compared to Part 1 (*P* = .25). Historical controls had significantly better relapse-free survival (*P* = .0007).

**Conclusion:**

In children with SDNS, this novel therapy with CB-MSCs was safe but failed to improve relapse-free survival 6 months after IS withdrawal.

## Graphical Abstract

**Graphical Abstract:**
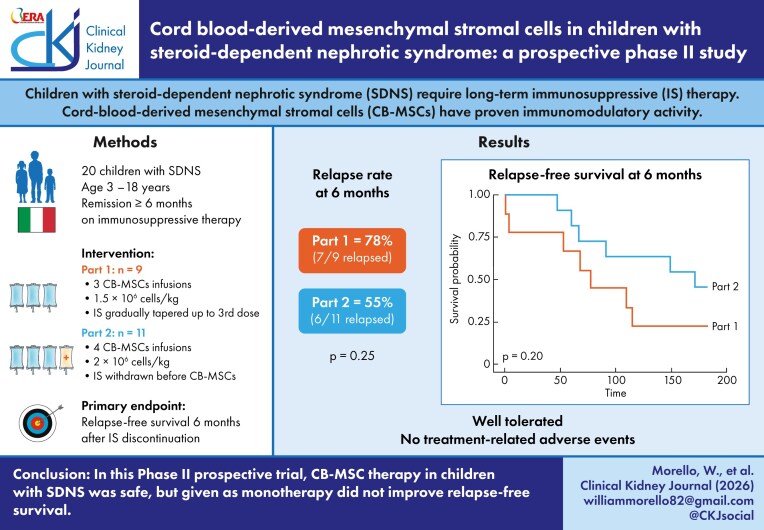

KEY LEARNING POINTS **What was known:**

- Children with steroid-dependent nephrotic syndrome (SDNS) require prolonged immunosuppressive therapy, which carries significant toxicity and impacts quality of life.
- Mesenchymal stromal cells (MSCs) have demonstrated immunomodulatory effects in preclinical models and clinical studies of immune-mediated diseases.
- Cord-blood-derived MSCs (CB-MSCs) were proven to be safe in children with steroid-resistant nephrotic syndrome, but their potential to maintain remission after immunosuppression withdrawal in children with SDNS was unknown.

**This study adds:**

- A novel therapy with CB-MSCs was safe but did not improve relapse-free survival following IS withdrawal in children with SDNS.
- Modifying the timing and dosage of CB-MSC administration did not significantly alter outcomes compared to the initial protocol or historical controls.
- These findings challenge the clinical utility of MSCs in this setting and highlight the limitations of their immunomodulatory effect in SDNS.

**Potential impact:**

- CB-MSC therapy should not currently be used to replace standard IS treatment in children with SDNS.
- This study informs clinicians and researchers about the limitations of CB-MSC-based strategies in this population, guiding future research priorities.
- Alternative approaches, including MSC-derived products or combination strategies, may be needed to improve outcomes in SDNS.

## INTRODUCTION

Idiopathic Nephrotic Syndrome (INS) is the most common glomerular disease in children, characterized by the triad of nephrotic-range proteinuria, hypoalbuminemia, and edema [1]. Although its pathogenesis remains incompletely understood, there is growing evidence supporting a central role of immune dysregulation [2, 3]. The mainstay of treatment is corticosteroids; however, ∼50% of patients develop frequent relapses or become steroid-dependent (SDNS), requiring prolonged steroid courses or additional immunosuppressive agents (IS) [4], which carry a significant burden of toxicity and negatively impact quality of life [5].

Mesenchymal stromal cells (MSCs) are multipotent, non-hematopoietic stem cells, which can be isolated from various tissues and differentiate into multiple lineages [6]. Beyond their regenerative potential, MSCs exhibit potent immunomodulatory properties, especially when stimulated by pro-inflammatory cytokines such as interferon (IFN)-γ and tumor necrosis factor-α [7]. In recent years, efforts by several research groups affiliated with cord blood (CB) bank networks [8] have focused on optimizing the isolation and expansion of CB-derived MSCs (CB-MSCs), refining their phenotypic characterization and therapeutic potential, key factors for advancing new clinical approaches in cellular therapy [9–13]. Our group has contributed to this field by advancing good manufacturing practice (GMP)-compliant manufacturing protocols and characterizing CB-MSCs for clinical use essential for the safe and effective translation into clinical applications [14–16]. We previously demonstrated the safety of CB-MSCs in a prospective, open-label, single-arm phase I–II pilot study involving children with multi-drug resistant INS [17], which also suggested a potential immunomodulatory effect in pediatric immune-mediated kidney diseases.

Building on these findings, we now aimed to assess the efficacy of CB-MSCs in modulating the immune dysregulation underlying INS, with the goal of reducing or discontinuing long-term IS therapy and its associated toxicities in children with SDNS.

## MATERIALS AND METHODS

### Study design

An open-label, single-arm, single-center, phase 2 trial with an adaptive second part was designed.

In the first part of the trial (Part 1), 11 children with SDNS were planned to be enrolled. After baseline clinical and laboratory screening, patients received three intravenous infusions of CB-MSCs (1.5 × 10^6^ cells/kg) on days 0, 14, and 21. The IS treatment was gradually tapered off with complete withdrawal after the third administration. An interim analysis was planned *a priori* at the end of Part 1, with a pre-defined efficacy threshold of a remission rate >60% at 6 months. If this threshold was not met, an adaptive phase (Part 2) would be implemented.

Part 2 followed the same inclusion criteria and sample size as Part 1, but with a modified IS tapering strategy. Ongoing IS treatment was rapidly reduced during the 2 weeks preceding enrollment, and fully withdrawn at least 24 h before the first infusion, to potentially enhance CB-MSCs biological activity [18, 19]. The only exception was tacrolimus, which was tapered and discontinued after the third infusion, as in Part 1. Patients received CB-MSCs at an increased dose of 2 × 10^6^cells/kg. Children who remained in remission at the first follow-up received a fourth infusion (booster dose). The procedures, follow-up and rescue therapies were identical to those in Part 1.

**Figure 1:**
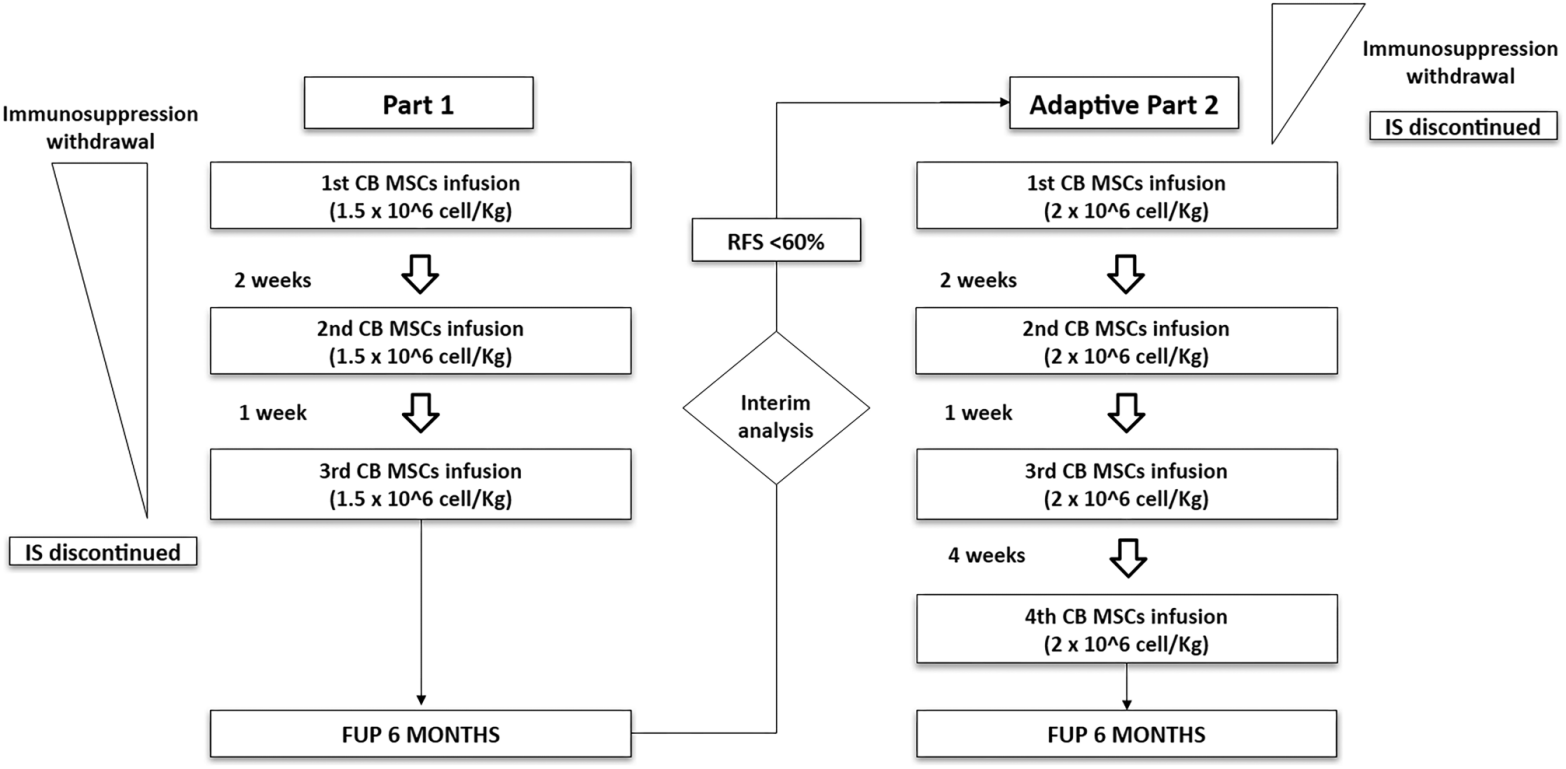
Study design. In Part 1, patients received three intravenous infusions of cord blood–derived mesenchymal stromal cells (CB-MSCs, 1.5 × 10⁶ cells/kg) on days 0, 14, and 21. Immunosuppressive therapy (IS) was tapered and withdrawn after the third infusion. An interim analysis assessed remission rate at 6 months, with a pre-defined threshold of >60%. If remission was ≤60%, the adaptive Part 2 was implemented. In Part 2, IS was rapidly tapered and stopped ≥24 h before the first infusion. CB-MSC dose was increased to 2 × 10⁶ cells/kg on days 0, 14, and 21. Patients in remission at first follow-up received a fourth (booster) infusion. Follow-up and rescue therapies were identical in both parts. CB-MSCs, cord blood–derived mesenchymal stromal cells; IS, immunosuppressive therapy; RFS, relapse-free survival.

A schematic flow diagram of the study design for both Part 1 and Part 2 is provided as Fig. 1.

### Study population

Inclusion criteria were as follows: (1) clinical diagnosis of SDNS; (2) age between 3 and 18 years; (3) disease remission for at least 6 months maintained by chronic IS therapy; (4) absence of proteinuria [urinary protein-to-creatinine ratio (uPr/uCr) <0.2 mg/mg]; (5) estimated glomerular filtration rate (eGFR) ≥70 ml/min/1.73 m^2^; (6) written informed consent from parents or guardians and assent from the child, when possible.

### Study drug

The investigational medicinal product was an advanced therapy medicinal product, made of MSCs derived from umbilical CB for allogeneic use. The product was developed and controlled at the institutional GMP-authorized Unit “Cell Factory” of Fondazione IRCCS Ca’ Granda, Ospedale Maggiore Policlinico in Milano (N° aM–11/2023). CB-MSC GMP production was performed as already described [14, 17]. Before administration, cells were thawed with a point-of-care dry-thawing device (Barkey Plasmatherm, Leopoldshöhe, Germany), then resuspended vol: vol in a saline solution with 10% HSA and Anticoagulant Citrate Dextrose Solution, Solution A (ACD-A, Terumo BCT, Tokyo, Japan) and reinfused as a final cell suspension suitable for intravenous administration. Details of CB-MSC treatment, including patient-level and aggregated data, are summarized in Table 2.

CB-MSCs were thawed at ambient temperature immediately before administration and delivered via peripheral intravenous injection, over 15 min, by trained personnel.

### Follow-up

Patients’ visits were planned at baseline, at each CB-MSC infusion, 2 weeks (follow-up visit 1, FUP1) and 6 weeks (FUP2) after the final infusion, and every 6 weeks thereafter, until 6 months after IS withdrawal. At each follow-up visit, uPr/uCr ratio was measured. Relapses were treated according to the Italian guidelines [20].

### Outcomes

The primary outcome was relapse-free survival at 6 months after the complete withdrawal of ongoing IS treatment with an efficacy threshold defined as a remission rate >60% at 6 months. Secondary outcomes included: relapse-free survival at 12 months, the reduction in the number of IS agent drugs at 12 months and the number and severity of CB-MSCs-related adverse events.

### Sample size calculation

The sample size was determined *a priori*. Assuming a remission rate of ≥60% would indicate clinical success and a rate of <20% would indicate treatment failure, with a type I error rate of 5% and a power of 80% power, a minimum of 10 patients was required for each part to assess efficacy against this threshold. To account for an anticipated dropout rate of ∼10%, 11 patients were planned to be enrolled in each study part.

### Post-hoc comparison to an historical control population

To better understand the clinical significance of the results, a post-hoc analysis was performed to compare the relapse rate in the study cohort with a historical control population. Data were retrospectively collected for all SDNS children, followed up at our unit, who were treated with IS drugs and had at least one previous attempt to discontinue the chronic IS treatment. The historical control population was selected through a retrospective review of medical records, applying the following inclusion criteria: (1) clinical diagnosis of SDNS; (2) age between 3 and 18 years; (3) disease remission for at least 6 months maintained by chronic IS therapy; (4) at least one previous attempt of IS treatment withdrawal; (5) follow-up >12 months after IS withdrawal. The relapse rates at 6 months were compared between the study cohort and the historical control population.

### Definitions

SDNS was defined according to the most recent guidelines as relapses occurring during steroid treatment or within 14 days after therapy discontinuation [20]. Chronic IS therapy was defined as continuous treatment with at least one steroid-sparing agent for ≥6 months. Remission was defined as a uPr/uCr ratio <0.2 mg/mg. Relapse was defined as the occurrence of uPr/uCr >2 mg/mg in at least two samples collected 3 days apart [21].

### Ethics

The study was approved by the local ethics committee (EudraCT No: 2018-001162-42) and by the national competent authorities [Agenzia Italiana del Farmaco, Authorization No: 2016-02364896]. Written informed consent was obtained from parents or legal guardians prior to the enrollment of children. All data were anonymized and de-identified before analysis.

### Statistical analysis

Categorical data are expressed as frequencies and proportions. Continuous data are expressed as means and standard deviations (SD) for normally distributed variables, or as medians with interquartile ranges (IQR) for non-normally distributed variables. Categorical variables were compared using the chi-square test or the Fisher’s exact test, as appropriate. Continuous variables were compared using non-parametric tests (Wilcoxon test for independent samples, Kruskal-Wallis test). Associations between predictor variables and continuous outcomes were assessed using simple or multiple linear regression models. For binary outcomes, simple or multiple logistic regression models were applied. Survival analyses were performed using the non-parametric Kaplan-Meier estimator and the log-rank test. Multiple regression survival analysis was performed using Cox proportional hazards models. A *P*-value <.05 was considered statistically significant.

Statistical analyses were performed using the open-source software R (R Core Team, 2022). *R: A language and environment for statistical computing*. R Foundation for Statistical Computing, Vienna, Austria. Available at: https://www.R-project.org.

## RESULTS

### Population

In the first part of the study (Part 1), 9 patients were enrolled (M: F ratio 7:2), with a median age at disease onset of 2.9 years (IQR 2.1–3.1) and a median age at study enrollment of 6.7 years (IQR 5.9–12.8). Throughout the course of the disease, patients had been treated with a median of 3 lines of IS drugs (IQR 2–4). At the time of enrollment, 8/9 (88.9%) were on combined IS therapy with a median duration of the ongoing IS treatment of 3.2 years (IQR 2.8–7.4).

In Part 2, we enrolled 11 patients (M: F ratio 7:4), with a median age at disease onset of 3.8 years (IQR 2.5–5.8) and a median age at study enrollment of 11.3 years (IQR 9.9–13.6). Patients had received a median of 2 lines of IS drugs (IQR 1.5–3). At the time of study enrollment, 5/11 (45.5%) patients were treated with combined IS therapy. The median duration of the ongoing treatment was 3 years (IQR 2.2–6.3). The clinical characteristics of the enrolled population are summarized in Table 1.

**Table 1:** Clinical characteristics of the enrolled population.

Clinical characteristics	Part 1 (*n* = 9)	Part 2 (*n* = 11)	*P* value
Age at enrollment in years, median (IQR)	6.6 (5.6–12.4)	11.3 (9.9–13.6)	.10
Gender, *N* (%) Males Females	7 (77.8%) 2 (22.2%)	7 (63.6%) 4 (36.3%)	.64
Previous lines of IS treatments, median (IQR)	3 (2–4)	2 (1.5–3)	.10
Current treatment, *N* (%) Monotherapy Combined therapy	1 (11.1%) 8 (88.9%)	6 (54.6%) 5 (45.4%)	.20
MMF CSA MMF + CSA MMF + FK	1 (11.1%) 1 (11.1%) 3 (33.3%) 4 (44.4%)	6 (54.6%) – 1 (9.1%) 4 (36.3%)	.14
Time between last relapse and IS withdrawal in months, median (IQR)	24.9 (13.4–27.8)	20.5 (13.5–27.3)	.82

IS, immune suppression; MMF, mophetil mycophenolate; CSA, cyclosporine-A; FK, tacrolimus; IQR, interquartile range.

### CB-MSC therapy

In Part 1, patients received three CB-MSC intravenous infusions on days 0, 14, and 21. The median dose was 1.7 × 10^6^ (1.4–2.0) MSC/kg of body weight. In Part 2, all patients received the initial three CB-MSC infusions and 9 children, who maintained remission at FUP1, received the fourth CB-MSC infusion. The mean dose was 1.80 × 10^6^ (1.7–2.5) MSC/kg of body weight. The median purity was 97.3 ± 2.7 with a median CD45^+^ contaminants of 0.6 ± 0.5 and a viability of 92.7 ± 4.6 in both groups. All products were confirmed to be sterile, mycoplasma-free, with a level of endotoxins below the acceptable limit of 0.25 EU/ml (Table 2).

**Table 2:** Per-patient summary of CB-MSC treatment by study phase.

Study phase	Patient code	*n* Infusions	Dose (×10⁶/kg) Mean (SD)	% CD90⁺/CD105⁺/CD45⁻ Mean (SD)	% CD45⁺ Mean (SD)	% PI⁻ Mean (SD)
Part 1	RACE_01	3	1.6 (0.0)	97.5 (3.6)	0.3 (0.5)	95.0 (0.6)
	RACE_02	3	1.7 (0.0)	99.0 (0.0)	0.1 (0.0)	93.9 (0.0)
	RACE_03	3	1.7 (0.0)	99.4 (0.3)	0.7 (0.4)	90.3 (8.2)
	RACE_04	3	1.4 (0.0)	99.2 (0.0)	0.5 (0.0)	95.1 (0.0)
	RACE_06	3	1.6 (0.0)	99.9 (0.0)	0.0 (0.0)	88.5 (0.0)
	RACE_07	3	1.8 (0.0)	99.2 (0.0)	0.0 (0.0)	93.5 (0.0)
	RACE_08	3	2.0 (0.0)	99.2 (0.0)	0.5 (0.0)	95.1 (0.0)
	RACE_10	3	1.6 (0.0)	98.8 (1.0)	0.0 (0.0)	92.5 (3.3)
	RACE_11	3	1.9 (0.0)	99.3 (0.9)	0.0 (0.0)	90.2 (4.0)
Part 2	RACE_12	4	1.7 (0.0)	98.6 (0.4)	0.6 (0.1)	95.3 (0.3)
	RACE_13	4	1.9 (0.0)	98.5 (0.6)	0.6 (0.5)	91.6 (2.9)
	RACE_14	4	1.8 (0.0)	99.2 (0.0)	0.0 (0.0)	93.5 (0.0)
	RACE_15	4	1.9 (0.0)	98.3 (0.0)	1.6 (0.0)	94.0 (0.0)
	RACE_16	3	1.8 (0.0)	98.4 (0.5)	1.0 (0.5)	94.3 (0.8)
	RACE_17	4	1.8 (0.0)	98.8 (0.5)	1.1 (0.8)	90.1 (2.7)
	RACE_18	4	2.0 (0.0)	98.0 (0.0)	1.1 (0.0)	89.3 (0.0)
	RACE_19	4	2.2 (0.0)	98.5 (0.0)	1.4 (0.0)	96.0 (0.0)
	RACE_20	4	1.9 (0.0)	97.9 (0.0)	1.9 (0.0)	92.4 (0.0)
	RACE_21	3	2.1 (0.0)	98.5 (0.0)	1.4 (0.0)	96.0 (0.0)
	RACE_22	4	2.1 (0.0)	98.8 (0.6)	1.3 (0.9)	93.2 (2.2)

Patient-level data are shown as mean ± SD for all infusions for each patient. The number of total CB-MSC infusions per patient is indicated. Part 1 and Part 2 are shown separately to reflect the study design. CB-MSC, cord blood–derived mesenchymal stromal cells; SD, standard deviation; PI, propidium iodide.

### Follow-up

All patients completed the 6-month follow-up period, and all were further followed up to 12 months.

### Primary outcome

In Part 1, 7/9 (77.8%) patients relapsed in the first 6 months after CB-MSCs administration and IS treatment withdrawal, while 2/9 patients remained in remission.

Since the primary endpoint was not reached, Part 1 of the study was stopped earlier and Part 2 was implemented as per the study plan.

**Figure 2:**
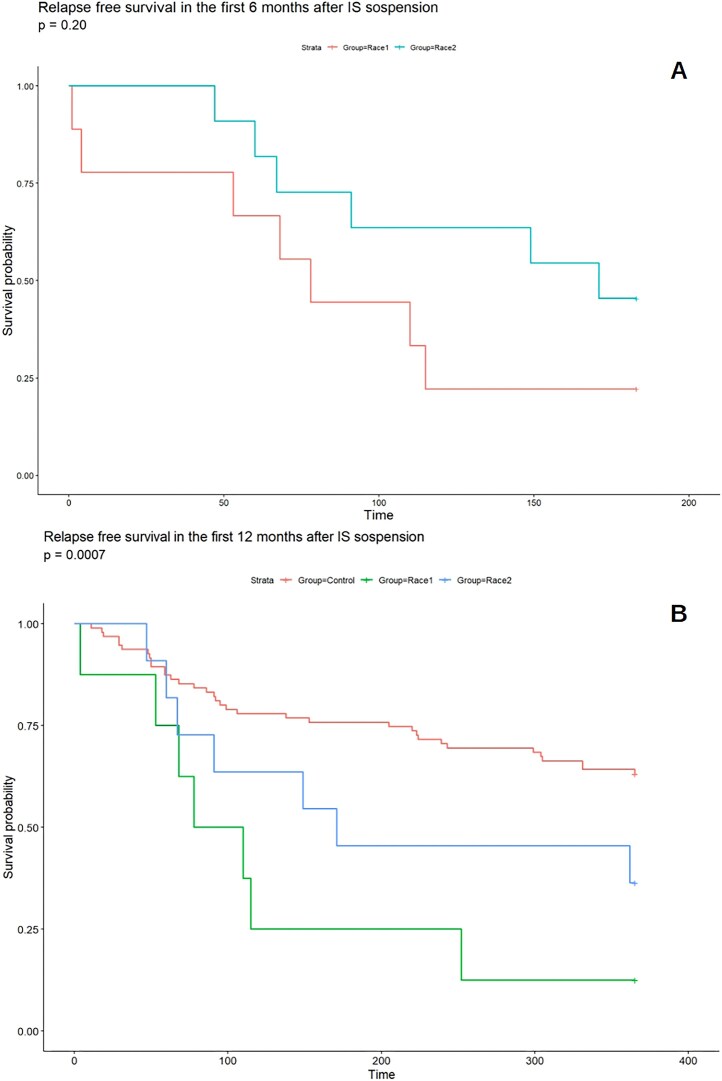
Relapse-free survival during the first 6 months after discontinuation of immunosuppressive therapy (IS). Data are shown for Part 1 and Part 2 of the study (A), and compared with the historical control population (B). Part 2 included protocol modifications, including a different tapering schedule of immunosuppressive drugs, an increased CB-MSC dose, and a fourth infusion (see Methods for details).

In Part 2, 9/11 enrolled patients were still in remission at the first follow-up visit and received the booster fourth CB-MSCs infusion. At the 6-month follow-up, 5/11 patients maintained stable remission, while a relapse was reported in 6/11 (54.5%). No statistically significant difference in relapse-free survival at 6 months after baseline IS treatment withdrawal was found between the two parts of the study (*P* = .2) (Fig. 2A).

Considering the entire sample, 13 of 20 patients relapsed within the first 6 months. The expected relapse-free survival at 6 months was therefore 35%. After adjusting for potential confounders in a Cox proportional hazards regression model, the difference between the two groups remained non-significant (*P* = .25). In this model, the only significant predictor of relapse-free survival was the time from last relapse before IS suspension to IS suspension (*P* = .01). Age at onset, age at IS suspension, and gender were not significant in this model.

### Secondary outcomes

In Part 1, at the 12-month follow-up visit, a relapse was reported in one additional patient, with a final relapse rate of 88.9%. IS treatment was restarted in seven relapsed patients at a median of 73 days after discontinuation of maintenance IS therapy (IQR 40.75–111.25).

In Part 2, at the final follow-up, 12 months after withdrawal of maintenance immunosuppression, 7/11 (63.6%) patients had experienced at least one relapse, and among them, 3/7 (42.9%) had multiple relapses. Six relapsed patients restarted maintenance IS therapy after a relapse at a median of 88 days after treatment withdrawal (IQR 72.5–148.5) with no significant difference compared to Part 1 (*P* = .49). Among these six patients, four switched from combination therapy to monotherapy, while two continued their previous treatment regimen unchanged. As a result, 5/11 (45.4%) patients remained free of immunosuppression at 12 months after discontinuation, while an overall reduction in treatment burden was achieved in 8/11 (72.7%) patients.

**Figure 3:**
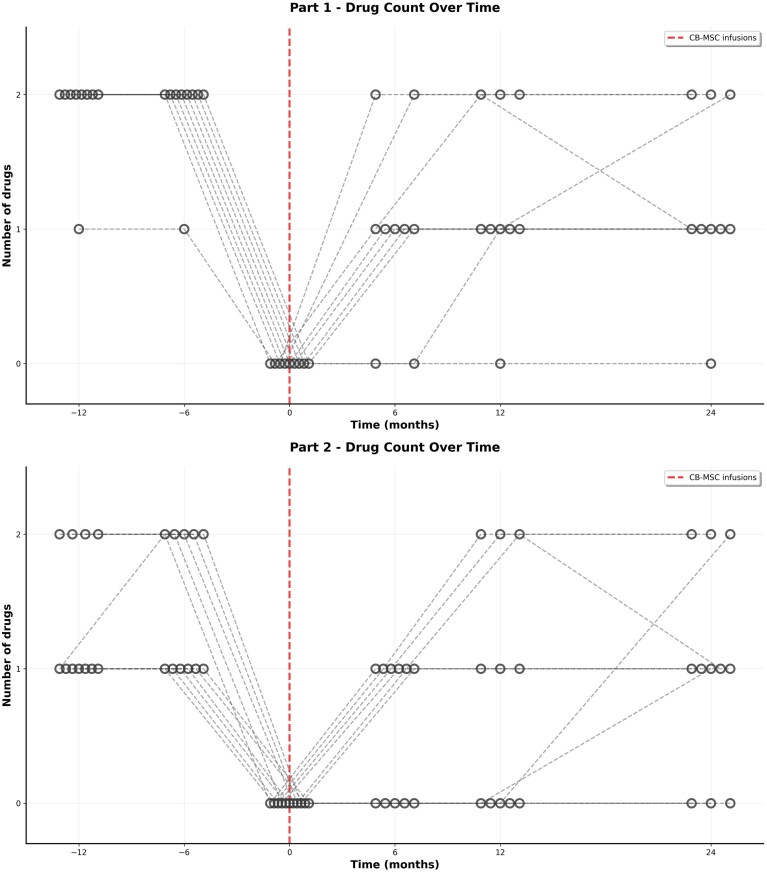
Number of immunosuppressive drugs administered over time in both patient cohorts. Each line represents an individual patient’s trajectory, with points indicating drug counts at each time point. Per protocol, immunosuppressive therapy was discontinued at the time of cord blood–derived mesenchymal stromal cell (CB-MSC) infusions (red dashed line). The plot shows trends in immunosuppressive therapy before and after CB-MSC administration.

A comparison between the number of ongoing IS drugs before treatment withdrawal after CB-MSCs therapy showed no statistically significant difference in Part 1 (mean difference = −0.33, *P* = .30). However, a significant reduction was documented in Part 2 (mean difference = −0.73, *P* = .02). Extended follow-up data were available for all patients. Figure 3 summarizes the number of IS drugs used in the study population from 12 months before to 24 months after treatment.

CB-MSC infusions were well tolerated, and no treatment-related adverse events were observed in either phase of the study (Supplementary Table S1). Safety was systematically monitored in all participants.

### Post-hoc analysis: comparison with a historical reference cohort

From a review of all digital medical records, 95 SDNS patients (M: F ratio 56:39) were selected as a historical reference cohort. All of these patients had undergone at least one previous attempt at IS treatment discontinuation, with the last attempt occurring at least 12 months prior to data collection. The age at the time of IS tapering and the time between the last relapse and IS withdrawal were not significantly different between the two study groups and this reference population (*P* = .13 and *P* = .64, respectively). However, all patients in the reference cohort were on a single IS agent at the time of tapering, and the tapering period was longer compared to the study groups, in accordance with standard clinical practice (*P* < .001).

In the reference cohort, 23/95 (24.2%) patients relapsed within 6 months of stopping treatment, while 35/95 (36.8%) relapsed within 12 months. The 6- and 12-month relapse rates were significantly lower in the reference cohort compared to both study groups (*P* < .001), with a significantly longer relapse-free survival (*P* < .001) (Fig. 2B). In a Cox multiple regression model, a shorter time from relapse to discontinuation and a younger age at treatment withdrawal were significantly associated with the risk of subsequent relapses (*P* = .005 and *P* = .02, respectively). However, the hazard ratio (HR) for relapses remained significantly higher in both study groups compared to the historical reference cohort, even after adjusting for these two potential confounders (Group 1 HR 5.7, 95% CI 2.3–14.2; *P* < .001; and Group 2, HR 3.3, 95% CI 1.3–8.6; *P* = .01).

## DISCUSSION

In this prospective, open-label, adaptive, phase 2 study, we evaluated the efficacy of a novel immunomodulatory therapy using CB-MSCs in maintaining remission after IS withdrawal in children with SDNS. A total of 20 children received at least three CB-MSC infusions, and were followed for at least 6 months. The therapy was well tolerated, with no treatment-related adverse events. However, most patients experienced disease relapse after discontinuation of the ongoing IS therapy, resulting in a relapse-free survival lower than anticipated, and inferior to that observed in the historical reference cohort.

MSCs are multipotent, non-hematopoietic stem cells with established immunomodulatory properties, including inhibition of B- and T-cell proliferation, enhancement of regulatory T-cells, and suppression of proinflammatory cytokines production [22]. These biological effects make MSCs a promising therapeutic option for several immune-mediated diseases [23]. Therefore, there was a strong rationale for their application in children with SDNS, the most severe form of steroid-sensitive INS, which is characterized by immune dysregulation [2], and associated with significant treatment-related morbidity, and impaired quality of life [5]. Previous clinical studies have explored the use of MSCs in other immune-mediated kidney diseases, particularly lupus nephritis, with mixed results. While some open-label studies have shown promising outcomes [24, 25], a randomized controlled trial reported no significant benefit [26].

The primary objective of this study was to test whether MSC therapy could maintain clinical remission in the absence of concomitant immunosuppression. For this reason, IS therapy was intentionally discontinued following MSC administration, allowing a direct evaluation of MSC efficacy while simultaneously reducing drug-related toxicity. The study was designed in two sequential parts, with an adaptive treatment modification implemented in case of inefficacy. The interim analysis of children treated in Part 1 showed a relapse-free survival at 6 months of 22%, much lower than the pre-defined efficacy threshold (60%). In light of the most recent insights from the literature, the treatment protocol was modified for Part 2. CB-MSC therapy was administered after the discontinuation of all IS agents, except tacrolimus, to avoid the potential negative interaction between CB-MSCs and IS drugs [18, 19]; the dose of CB-MSC was slightly increased to 2 × 10^6^/kg to explore a dose-efficacy correlation, and an additional fourth dose was added to potentially prolong the immunomodulatory effect.

These changes were based on preclinical evidence suggesting that MSCs require exposure to a proinflammatory environment, particularly IFN-*γ*, to acquire full IS activity. *In vitro* and animal studies have shown that IFN-*γ* activates MSCs via induction of indoleamine 2,3-dioxygenase, enabling T and NK cell inhibition. Concomitant IS agents, such as mycophenolate mofetil, may weaken this activation and reduce MSC proliferation and function, while tacrolimus may have a neutral or even synergistic effect [18, 27].

Despite these modifications, relapse-free survival in Part 2 was 45%, only marginally higher than in Part 1 and not statistically different (*P* = .2, log-rank test), and lower than in the historical control cohort. We acknowledge the limitations of historical comparisons, including potential selection bias. The historical cohort included only patients in stable remission on a single IS agent who successfully initiated IS tapering and excluded early relapsers, which may partly explain their better outcomes. For this reason, the historical comparison was included as a post hoc contextual analysis, while the main conclusions rely on predefined endpoints within the treated cohort.

At 12 months, a reduction in IS burden was observed in Part 2. However, this finding must be interpreted cautiously, as it does not account for the typical long-term escalation of IS therapy seen in SDNS, where additional IS agents are often added sequentially, nor for the natural tendency of disease activity to decrease over time in children with idiopathic nephrotic syndrome [1]. Morever, a longer follow-up up to 24 months did not show a meaningfully impact of CB-MSCs on long-term treatment burden. Only a randomized controlled trial could confirm this effect; however, due to ethical concerns and the rarity of the disease, such a design was not feasible.

Our findings are consistent with those of a recent study using autologous bone marrow–derived MSCs in children and adults with SDNS [28], in which all patients experienced relapse despite gradual IS tapering. Although that study reported a reduction in relapse frequency in children compared with their prior clinical history, the prolonged tapering of IS and absence of a control group complicate interpretation. Differences in patient severity, MSC source, study design, and concomitant immunosuppression likely account for discrepancies between studies and highlight the challenges in assessing MSC efficacy in SDNS.

Despite being in stable remission at enrollment, seven of nine patients in Part 1 and 6 of 11 patients in Part 2 experienced relapse, highlighting the limited efficacy of CB-MSCs in maintaining remission after withdrawal of IS therapy. Taken together, our results suggest that CB-MSCs, when used without concomitant immunosuppression, are insufficient to maintain remission in most children with SDNS. However, these findings do not exclude a potential adjunctive role for MSCs under different conditions. Future studies should focus on strategies to enhance MSC efficacy, such as preconditioning MSCs to inflammatory stimuli, improving their persistence and function *in vivo*, or exploring MSC-derived extracellular vesicles, which may concentrate immunomodulatory mediators [29, 30]. Given the transient nature of MSC immunomodulatory effects observed in multiple disease settings, alternative dosing schedules or maintenance strategies may also warrant investigation, although our data suggest that repeated infusions over a short period are unlikely to provide sustained benefit.

This study has several limitations, including the small sample size, lack of a randomized control arm with historical comparisons, absence of detailed immunophenotyping, and relatively short follow-up. Nevertheless, enrolling 20 children with SDNS represents a significant effort in a rare disease, and the consistency of outcomes across patients, with most relapses occurring within 6 months, strengthens the reliability of our conclusions.

In conclusion, the use of CB-MSC therapy in children with SDNS was safe but resulted in lower-than-expected relapse-free survival, with most patients relapsing after the discontinuation of ongoing IS therapy. Both treatment schedules yielded similar outcomes, with no significant difference in relapse-free survival between the two parts, and a higher relapse rate compared to a historical control population. Despite a biological rationale supported by preclinical evidence, our clinical results do not support the use of CB-MSCs as a standalone disease-modifying therapy in children with SDNS. Given the lack of efficacy signal, along with ethical and logistical challenges of conducting larger studies in this rare population, a randomized controlled trial does not appear justified at present. However, the exploration of alternative MSC-derived strategies may offer potential options for future research in this area.

## Supplementary Material

sfag179_Supplemental_File

## Data Availability

The data underlying this article are available in the article and in its online supplementary material.
