# Supplementary material for: Cord blood-derived mesenchymal stromal cells in children with steroid-dependent nephrotic syndrome: a prospective phase II study
Source: Clin Kidney J. 2026 May 30;19(7):sfag179. doi: 10.1093/ckj/sfag179 (PMC13325487; doi:10.1093/ckj/sfag179)
Supplement: sfag179_Supplemental_File [file sfag179_supplemental_file.docx]

**Supplementary Table 1 – Treatment related adverse events recorded during the study.**

| Phase | Number of patients | Any treatment-related AEs | Grade ≥3 AEs | Serious AEs |
| --- | --- | --- | --- | --- |
| Part 1 | 9 | 5 | 0 | 0 |
| Part 2 | 11 | 2 | 0 | 0 |

**All patients were monitored for adverse events throughout the study. AE, adverse event.**
